# Multi-echo single-shot EPI for hyperpolarized 13C cardiac metabolic imaging of small animals

**DOI:** 10.1186/1532-429X-15-S1-P217

**Published:** 2013-01-30

**Authors:** Andreas Sigfridsson, Kilian Weiss, Lukas Wissmann, Julia Busch, Marcin Krajewski, Darach O h-Ici, Michael Batel, Georgios Batsios, Sebastian Kozerke

**Affiliations:** 1 Biomedical Engineering, ETH Zurich, Zurich, Switzerland; 2 Congenital Heart Disease and Pediatric Cardiology, Deutsches Herzzentrum Berlin, Berlin, Germany; 3 Imaging Sciences and Biomedical Engineering, King's College London, London, UK; 4 Physical Chemistry, ETH Zurich, Zurich, Switzerland

## Background

Cardiac metabolic imaging based on hyperpolarized 13C-labeled pyruvate shows great potential for assessing the metabolic changes that the heart undergoes during ischemia [1]. Rodent animal models offer unique opportunities to study ischemic processes, however, methods based on spectral-spatial excitation [2] of the individual metabolites is challenging due to the large minimal slice thickness that can be achieved with available gradient systems. A thick slice introduces both signal dephasing over the slice and errors due to partial volume effects.

In this work, we explore multi-echo measurements using single-shot echo-planar imaging (EPI) readouts for thin slice dynamic cardiac metabolic imaging of small animals.

## Methods

Healthy female Wistar rats (200g) were scanned in a 9.4T horizontal bore Bruker MRI system. A home-built multi-sample dissolution DNP system was used to hyperpolarize pyruvate [3]. During image acquisition, 2ml 45mM 13C-labeled hyperpolarized pyruvate was injected in the tail vein. The pulse sequence consisted of 7 echoes separated by 383us, each triggered to 80ms after the R-wave of the ECG. With an RR-interval of ~180ms, a temporal resolution of ~1.3s was obtained. For each image, a 4mm short axis slice was excited with a flip angle of 8 degrees, and a 60x60mm2 FOV was read out using a single-shot EPI-readout with a matrix of 30x30. Images for each metabolite were then reconstructed and separated into lactate, pyruvate hydrate, alanine, pyruvate and bicarbonate using a multi-point Dixon technique [4,5]. The reconstruction included correction of EPI gradient delays, B0 offset, chemical shift displacement and separation.

## Results

**Figure 1:**
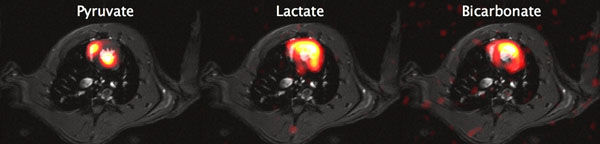
Metabolic images of pyruvate (left), lactate (center) and bicarbonate (right) as a sum over the signal bolus. Signal loss in the posterior wall of the heart is due to the sensitivity of the surface coil lying on the chest of the rat. The thin slice excitation reduces the impact of dephasing due to magnetic field inhomogeneities across the slice as well as partial volume effects. The result is a higher effective in-plane resolution.

**Figure 2:**
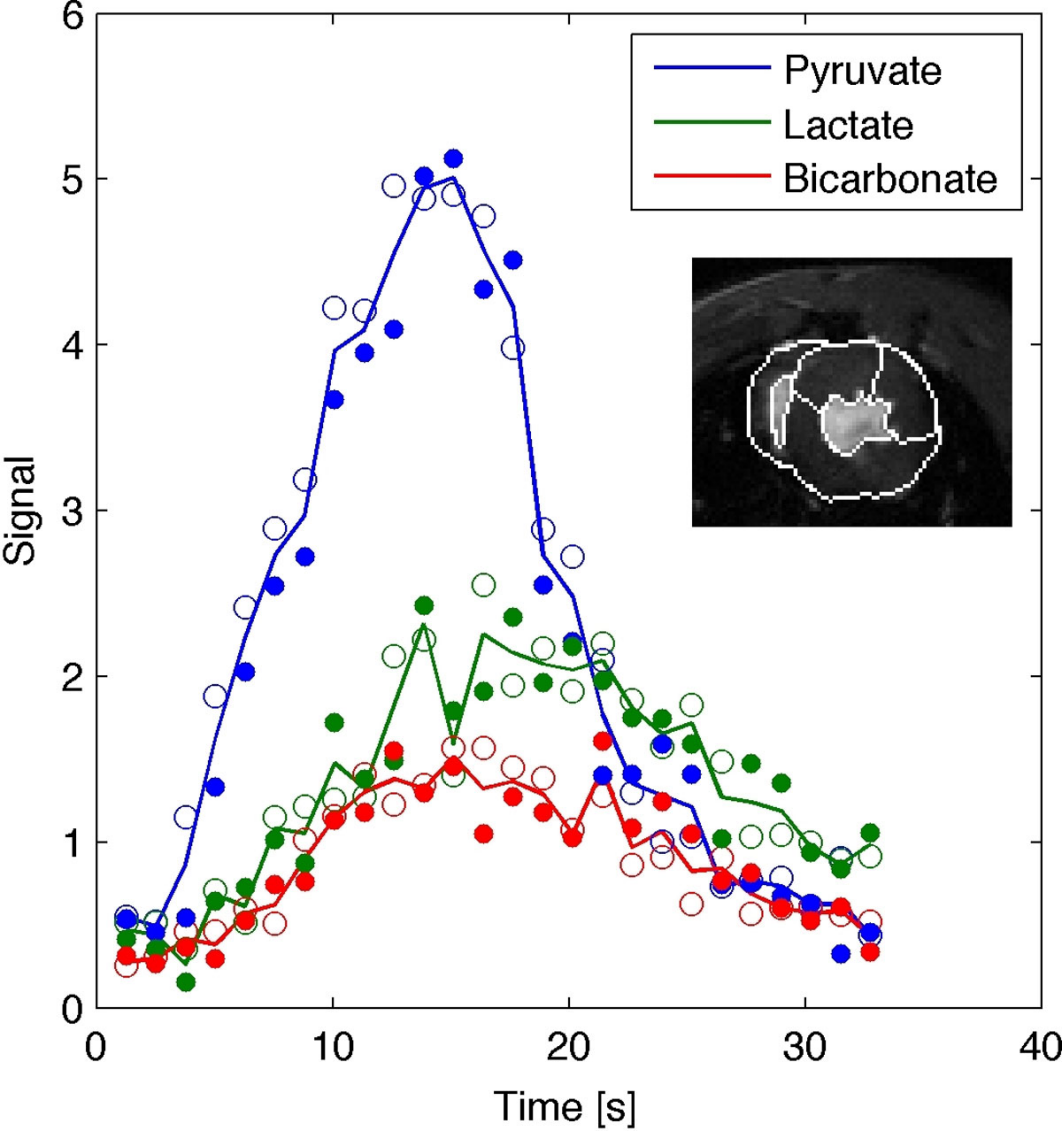
Time curves of pyruvate, lactate and bicarbonate in the myocardium. Open circles show antero-septal segment and closed circles show antero-lateral segment, whereas the full line shows both segments combined. The segments analyzed are shown in the short-axis inset.

Images for the metabolites are shown in Figure 1. Time curves for the metabolites in two segments of the myocardium are shown in Figure 2.

## Conclusions

By using the multi-echo technique, the requirements on the gradient system performance are relaxed. In contrast to using a spectral-spatial excitation, a simple slice excitation can be used that allows for thinner slices. This is especially important in small animal imaging. Due to the thin slices, the dephasing and partial volume effects are significantly reduced, and the effective in-plane resolution is improved. This is especially important in separating the signal from pyruvate between myocardium and blood pool when using kinetic modeling. The high in-plane resolution is of importance for metabolic imaging after selective occlusion of one coronary artery, and for studies of the metabolic changes that the heart undergoes under controlled experiments in small animals.

## Funding

Marie Curie Intra European Fellowship

Swiss National Science Foundation
